# Remote sensing and feminist critique: Reappropriations of sensing across distance

**DOI:** 10.1177/26349825241283838

**Published:** 2024-10-24

**Authors:** Lilian Kroth

**Affiliations:** University of Fribourg, Switzerland

**Keywords:** Remote sensing, distance, feminism, critical theory, access

## Abstract

Remote sensing technologies and Geographic Information Systems (GIS) have sparked diverse conceptual and critical responses, with feminist approaches in particular undergoing notable transformations. This article traces and contextualizes these changes, emphasizing the re-evaluation of the concept of “distance” in feminist critiques of remote sensing (RS) and GIS from the 1990s onwards. It highlights a shift from outright rejection of GIS/RS technologies as tools of masculinist, positivistic science to a redefinition of remotely sensed information and imbricated understandings of distance and proximity. The article argues that researchers view issues of masculinism from markedly different perspectives and re-evaluate the role of technology-mediated seeing and imagining, resulting in a re-evaluation of physical distance in its relationship to infrastructure, access, and care. What has transformed is what it means to be “critical” *toward* as well as the possibility to be “critical” *with* remote sensing from a feminist point of view.

## Introduction

An exponentially increasing number of satellites are forming something akin to a metallic belt around planet Earth. Many of these remote sensors face down to Earth, making it possible to gather various sets of data from afar. Without their presence being necessarily felt, they function as “eyes in the sky” that can span wide distances. Remote sensing (RS) in its various forms, technologies, and histories has sparked responses and criticism from researchers in different disciplines. To sense remotely—that is, to gather data through sensors that exceed the range of the lived experience of the human eye or the human senses in general^1^—is a prolific subject of research for political history, geography, science and technology studies, and critical theory (Aday and Livingston, 2009; Bennett et al., 2022; Cosgrove, 2001; Gabrys, 2019; Gärdebo, 2019; Kurgan, 2013; Parks, 2009). Critical inquiries into RS send us to military and logistical history but also to questions of scientific (un)certainty, scale, abstraction, alienation, domination, and objectivity. Early critiques, like Hannah Arendt’s 1958 work, *The Human Condition*, where she describes Sputnik as causing not only an alienation from the world (*Weltentfremdung*) but also from the Earth (*Erdentfremdung*) (Arendt, 2016), exemplifies various critical perspectives focused on humanist questions amid rapid military and technological advancements. In more recent decades, critical perspectives on RS and GIS (Geographical Information Systems) have proliferated, especially tackling the imbrication of these technologies in systems of classed, gendered, and racialized domination.^2^

This article shows how feminist critique of RS has witnessed significant transformations over recent decades. Feminist critiques have historically and conceptually been entangled with RS, here focused on the period after the launch of Sputnik in 1957. This includes whole Earth images of the 1960s and “70s (famously ‘Blue Marble” in 1972) up to nowadays’ intensified usage of satellite composite imagery in public and economic domains. RS as a set of technologies connected to practices and imagery has been critiqued by feminist scholars from a variety of angles and with notable changes over time. While some feminist researchers critique on-the-ground methods for their assumption of accessibility, others critique RS for its detached distance. Meanwhile, yet others problematize these latter critiques for being external to any practical implementation and, therefore, themselves “too distant.” In these different critiques, “distance” matters on different levels, which this article addresses through an integrated historical and conceptual review.

As feminist thought has evolved, with technology becoming more ubiquitous, climate crises growing more urgent, and the gendered and colonial biases of ethnography becoming more apparent, the concept of distance has shifted from being viewed with suspicion to being regarded as potentially productive within feminist circles. It will be argued that shifts in feminist critiques of RS involve heterogeneous theoretical traditions and forms of critical inquiry, and that the relationship between feminist critique and RS practices can be understood as reciprocally impactful. Through an inquiry that integrates historical and conceptual perspectives, it will be demonstrated that feminist critiques of RS/GIS have brought about remarkable shifts in the evaluation of concepts of distance. A review of these debates as a history of critical practice will address heterogeneous positions and the theoretical backgrounds that have inspired them, thereby not only tracing conceptual developments but also contextualizing their genesis. The following investigation will attempt to undermine a dichotomy between internal and external critiques of RS and show that other heuristics focusing on tropes of distance, gaze, and access prove to be more fruitful.

Starting off with approaches which emphasize RS’s implication in ways of seeing that are associated with masculinist science, as well as a masculinist perspective of dominance and control, I will discuss problematizations of such critiques advanced in the 1990s and contrast them with other feminist positions, which focus on potentially productive aspects of distance and usages of RS/GIS. My questions include, how have different factors shaped and reshaped the demands for feminist critique of GIS and RS? How do feminist approaches to these technologies differ conceptually over time, and in what ways exactly? And how can we grasp the shifting role of “distance” as a practical and epistemological paradigm according to feminist approaches?

## Ecofeminist criticism of remotely sensed data, and the critique of criticisms

### Satellites, GIS, and masculinism

The relationship between sensing and distance is a topic that feminism has traditionally a lot to say about. By first giving a few examples of feminist critiques of GIS and RS in the 1990s, I will draw attention to some of their theoretical backgrounds and borrowings in ecofeminism and French theory. I will then turn toward problematizations of these early feminist critiques—in a way, the *critique of criticism*, leading to new engagements with RS and GIS.

Feminist critiques have significantly influenced how geographers assess the (male) gaze as a key framework for understanding subjectivities and landscapes. In this context, the contributions of scholars such as Gillian Rose are particularly noteworthy (Rose, 1993, 1995). Within a field broadly concerned with gendered forms of looking at environments through technologies, one significant line of feminist critique has scrutinized masculinist and positivist underpinnings as a general and indispensable part of RS and GIS (Bondi and Domosh, 1992; Curry, 1995; Garb, 1990; Roberts and Schein, 1995). Roberts and Schein, for example, assert that through the “view from above, we are establishing our own superiority and our domination of the scene”; we “imagine ourselves as separate from the view, situated somehow outside the data, and the view or its contents become ours to control and manage” (Roberts and Schein, 1995: 183). The authors call this form of gaze a “proprietorial objectivism” and claim that “the satellite’s way of seeing is that of the voyeur or even of the violator” (Roberts and Schein, 1995: 189).

RS, here, appears as an amalgamation of a positivist, masculinist, disembodied, scientistic, abstracted, and imperial project. In such ecofeminist criticisms of picturing the Earth through satellite visions, what is at stake is a “totalizing” view: part of this gaze generated through Earth imagery involves a relationship with the Earth and a desire to gain autonomy and independence from it. Other relatable positions in the 1990s take issue with the incapability to recognize women’s work on a domestic scale, which was not visible from the satellite’s perspective (Rocheleau et al., 1995). In all these aspects, it is argued, RS technologies as such have masculinist underpinnings.

A prime example of such a view is Yaakov Gerome Garb’s (1990) paper “Perspective or Escape? Ecofeminist Musings on Contemporary Earth Imagery.” Here, Garb negotiates an ecofeminist perspective of whole Earth images, clearly problematizing the imagery as a masculinist endeavor. Placing oneself into an outside in such a way would “create not only a physical distance but a corresponding psychic aloofness,” giving rise to “a sense of detachment and spectatorship”—a “distancing flavor” (Garb, 1990: 266–267). On various occasions, Garb refers to Susan Sontag’s writings on photography to grapple with the voyeuristic nature of “full disc” Earth images, ultimately remaining the “*magnum opus* of patriarchal consciousness” (Garb, 1990: 275). In pondering whether there might not be another way of relating or looking at these images, Garb insists that “we should check carefully whether we really want to view our relationship with the Earth through genderized lenses” (Garb, 1990: 277).

In her paper “The Gendered Eye in the Sky: A Feminist Perspective on Earth Observation Satellites” published in 1997, Karen T. Litfin also questions the social, philosophical, and gendered underpinnings of the planetary gaze (Litfin, 1997: 26). Just as Liftin notes, the “key catalyst of the remote sensing project” in the 1980s and 1990s were climate change debates (Litfin, 1997: 28), so climate change and ecology have remained a point of focus for critique in subsequent decades. Ecofeminist positions have unfolded as an influential voice in these earlier critiques. Without reducing the complexity of the ecofeminist tradition or overlooking the impact of a generalized pushback of ecofeminism by “third-wave feminism” (Thompson, 2006),^3^ what appears crucial is a widely shared concern with modernity’s gesture of dividing nature from the human sphere, and a co-emergence of the domination of nature and women (Merchant, 1980).

For this reason, ecofeminist positions have developed a sensitivity for registering the impact and consequences of such domination in different practices. Ecofeminists, according to Litfin, “would claim that the global gaze, by virtue of its position apart from and above nature, does violence to nature” (Litfin, 1997: 39). This gaze, which intends to maximize the “actual and felt distance,” is often connected to masculinist ideas of managing a posed problem or a “managerial impulse.” In reference to Joni Seager, Litfin draws critical attention to “the gendered division of labor, whereby men think about the environment and women care about it” (Litfin, 1997: 31, 43). I will return below to this implicit association of labor with distance and masculinity, as opposed to proximity and intimacy as ostensibly feminine modes of relation.

Although Litfin takes up ecofeminist critiques of objectification, her position is less firm as to how far these violent ways of looking and objectifying are necessarily inscribed into the technology. Still, she maintains a skepticism toward RS as a celebrated opportunity for global welfare, with questions that seem to motivate critical approaches to RS up until now: “Must we not be skeptical of a technology that promises so much? If celebratory discourses serve a masking function, then what might be said of the shadow side of remote sensing” (Litfin, 1997: 29)?

Before moving on to contestations and transformations of these feminist critiques, I would like to briefly outline some of the theoretical background to these early critiques of RS and GIS. Feminist critiques of RS in many cases coincide with 20th-century French theory, often assimilated under the label “poststructuralism” or “postmodernism.” A wide range of French thinkers and scholars, such as Gaston Bachelard, Paul Virilio, Michel de Certeau, and Henri Lefebvre, form major touchstones in these contexts. Themes such as the “totalizing eye” or “views from above” (Barthes, 1997; de Certeau, 1990), are widely taken up in feminist critiques of satellite imagery, whole Earth images, as well as GIS. For example, references such as Bachelard’s notion of an image’s “psychic state” has served ecofeminist approaches to Earth imagery (Garb, 1990), while Lefebvre’s concept of the production of space has been used to criticize GIS data (Roberts and Schein, 1995). Jacques Derrida is a further influence when it comes to RS’s “hauntological” affectivity (McCormack, 2010). The satellite’s role as a visual regime has furthermore been considered relatable to Foucault’s analyses of the panopticon as well as with his writings on the genesis of the gaze from the 17th century onwards (Nardon, 2007).

Elwood and Leszczynski argue that “most critical GIS origin stories” would build up on French theory rather than acknowledging the “direct take-up of feminist critiques of scientific objectivity and vision” (Elwood and Leszczynski, 2018: 2). This observation can be complemented by noting that various strands of French-language philosophy and French theory considerably influenced feminist approaches in this context. The reason for this is not that all of these authors explicitly bring feminist concerns to the fore; rather, it has to be understood in connection with the ascendancy of “French theory” in US humanities departments especially at this time, as well as with French theorists’ critical engagement with power, space, and ways of feeling, sensing, or being affected, which provides feminist critiques with a critical vocabulary of viewing from above or from afar.

### Feminist problematizations of feminist critiques

Some cited contributions from the collection *Ground Truth* (Pickles, 1995) are exemplary of feminist approaches whose critique sparked furor among GIS researchers who felt their work wrongly portrayed (Modarres, 1997; Openshaw, 1997; Schuurman and Pratt, 2002: 298). The volume sparked responses in defense of quantitative work through GIS, demanding feminist critique to be less abstract and more engaged with concrete suggestions and case studies (Openshaw, 1997). Schuurman and Pratt dedicate a paper to the culture of “critique” when it comes to feminist problematizations of GIS, arguing that many of the critiques of GIS “have aimed to demonstrate what is ‘wrong’ with this subdiscipline of geography rather than engaging critically with the technology” (Schuurman and Pratt, 2002: 291). Here, the argument against feminist critiques is that they remain “external,” and—in their epistemological motivation—unproductive.

A further set of responses has questioned whether these critiques offer viable alternatives to the dichotomies they address. On such a view, skepticism toward binaries on different fronts reproduces dichotomies of its own (Krylova, 2016; Lawson, 1995). This concerns, for example, descriptions of the “feminine landscape” and the “male abstract observer” who would try to “cut bonds” and gain independence and distance from the naturalized motherland.^4^ Victoria Lawson, for example, argues that some of the dualisms which feminist research seeks to undo are at times explicitly reinforced. For Lawson, the dualism between quantitative and qualitative research in feminist geography that has been particularly liable to reinforcing a markedly gendered distinction: quantitative methods are seen as “rigorous, objective, logical, and scientific (superior),” “whereas qualitative methods are designated as soft, subjective, irrational, and opinion (inferior)” (Lawson, 1995: 450–455). In reinforcing this dualism, the argument goes, feminist geographical research conflates “counting” with a “particular epistemology” and “technique” with a “philosophical position” (Bennett, 1985: 223; Lawson, 1995: 451).

## Shifting notions of the natural world: Nature, objectivity, situatedness

Very broadly, one might distinguish between two approaches to the feminist critique of RS (which can also intersect). On one hand, it can *seek to change practices* of gathering data remotely in the light of feminist critique—by addressing, for example, the research process, the backgrounds of people conducting the research, and the selection of research subjects. On the other hand, it can aim to *conceptualize RS as a practice*, and therefore change assumptions about what RS actually presents and in what terms it can be critically grasped. The second understanding of “critique” is more of an engagement with RS in conceptual terms, rather than a direct proposal for researchers with regard to how to gather data more critically. Nonetheless, both forms of critique have been historically important, if in different ways and on different levels.

Such a heuristic allows us to avoid the normative pitfalls of a distinction between feminist critique as either external/destructive/abstract or internal/productive/engaged. This latter division, which was widely used in feminist critiques of the late 1990s and early 2000s and is itself engaged in doing critique of GIS in a “more productive” manner, falls short as a framing for feminist critiques of GIS in general. It not only employs a normative framework, but also tends to depreciate epistemically motivated feminist critiques more generally. In the interests of providing a systematic account of feminist critiques of RS, there are good reasons to avoid boxing epistemological critiques as “external” and so paradoxically “distant” themselves or not constructive per se, and remain open to the possibility that “highly abstract,” conceptual or metaphorological interventions may in fact do work from “within.”

Against the backdrop of heuristics of critique, the following will discuss how feminist approaches reconsider the bases of their epistemological critiques, particularly in terms of biased relationships with “nature,” its own situatedness, and narratives of distance. Donna Haraway’s notion of the “godtrick of seeing everything from nowhere” is perhaps one of the most cited keywords in feminist critiques of the production of knowledge, the presentation and visualization of data, and RS. The metaphor first appears in her 1988 essay on situated knowledges, a text which has been broadly received in feminist scholarship across disciplines. What Haraway means by the “godtrick” is the illusion of infinite vision suggested by certain images—not, as it is sometimes assumed, the technological, artificial, or inhuman nature of such visions. Haraway insists on the “particularity and embodiment of all vision (although not necessarily organic embodiment and including technological mediation)”; and rather than turning away from mediated vision or the idea of objectivity, she seeks to dismantle such “tricks” of vision to reinvent “objectivity” accordingly (Haraway, 1988: 582). Haraway’s figure of the “modest witness” for technoscience suggests a situated and embodied attempt to reclaim technologies of vision for a feminist project (Haraway, 1997).

The epistemological critique of Haraway is key reference for the critical evaluation of data and images gathered and made from afar and has notably been related to the notion of “standpoint theory.” However, there is no necessary or direct passage between, on one hand, a standpoint in its socially situatedness and its partiality and, on the other hand, the critique of a perspective presented by a satellite. Although a direct translatability from standpoint theory into critiques of RS seems sometimes assumed, the frictionless connection is not entirely self-explanatory. Nevertheless, standpoint theory has given feminist scholars key concepts to investigate the (gendered) infrastructure around RS technologies and the (gendered) perspectives these facilitate in imagery.

Haraway often serves as a reference point in analyses of the authority of vision (as one of many senses) as part of a patriarchal regime of the “observer,” as also mentioned in the previous section on early feminist critiques. However, it has not often been acknowledged that Haraway’s criticism is not a plea *against* objectivity, nor against whole Earth images, but rather for a more aligned notion of objectivity in acknowledgment of its situatedness. Hence, her approach cannot be reduced to subjectivism, and it is for this reason that it also refuses an anthropocentric idea of experience.^5^ Readings of Haraway’s work have also been key to later differently configured critical engagements which reclaim feminist critique as a way of *situating* RS practices: a process not of dismissal, but of revelation and contextualization.^6^ With technological changes in RS, the arena of “objectivity” also seems to be changing. There is an insistence on RS’s situatedness, especially since the resolution of satellite images has increased and with it their seeming accuracy (Braun, 2021). Furthermore, apart from more general contestations of the satellite images’ objectivity as tied to a positive-masculinist view, in the face of “deep fake” imagery (Stănescu, 2022; Zhao et al., 2021), critical RS scholars stress the scrutiny of contexts and that “regardless of its solution, satellite imagery must be taken with a grain of salt” (Bennett et al., 2022: 733). Haraway’s idea of situatedness is therefore a persisting demand in RS—in fact, a contextualization and metaphorical “grounding” of RS.

Another example of just such an intervention is Haraway’s “cyborg,” whose significance on feminist understandings of science, data visualization, and the nature–body–technology nexus as a simultaneously “ontological” and “epistemological” contribution (Wilson, 2009) cannot be overstated. As a metaphor, Haraway takes up the concept that has first been suggested for the hybrid between man and machine in the 1960s and transfers it from the context of the military-industrial complex into a feminist context (Tollmann, 2022: 250–251). The transition away from the military context and the explicit entanglement of Haraway’s cyborg with “women’s anti-nuclear activism at the end of the Cold War era became largely eclipsed,” as Anna Feigenbaum argues, and would have to be reactivated to generate a critical understanding of contemporary cyborgs (Feigenbaum, 2015: 270). According to Haraway (2016), a

cyborg is a cybernetic organism, a hybrid of machine and organism, a creature of social reality as well as a creature of fiction. [. . .] The cyborg is a matter of fiction and lived experience that changes what counts as women’s experience in the late twentieth century. This is a struggle over life and death, but the boundary between science fiction and social reality is an optical illusion. (pp. 6–7)

Haraway marks her approach to the cyborg as one that starts from “histories, technologies, ecologies of the spacefaring NASA machine-organism-hybrids,” and the motivation was to decontextualize and reappropriate those cyborgs “to do feminist work in Reagan’s Star Wars times of the mid 1980s” (Haraway, 2004: 297).

The context of the 1980s is crucial here: Haraway’s concern was that feminism was at risk of falling into “back to nature” narratives that are incapable of responding to techno-scientific developments whatsoever. Haraway’s work remains skeptical toward latently hostile relationships with technology or aspirations of finding more “natural” ways of living beyond technologized environments (Tollmann, 2022: 248; Turner, 2006: 11–12); yet it also generates resistance toward “techno-strategic discourse” (Feigenbaum, 2015: 271). From the late 1980s onwards, she develops a theoretical toolkit which goes against the grain of the feminist mainstream of her contemporaries. With the implementation of technoscience into her conceptual framework, Haraway paves the way for less “naturalizing” forms of femininity, but also a less static understanding of nature and its relationship to the human and nonhuman—something that has been explicitly and emphatically taken up by Schuurman, for example, in her “cyborg manifesto for GIS” (Schuurman, 2002).

It is important to note that Haraway’s conceptualizations have been productive for the development of feminist philosophy of science which has, in turn, been important for recent feminist views on RS. Karen Barad’s concept of the “apparatus,” which they take over from Haraway and develop in their engagement with Niels Bohr is an instructive case. The “apparatus” in Barad’s terms is neither a concept for a static object nor a structure with a defined location, but a material-discursive reconfiguration of the world itself. As they write, “Apparatuses are not inscription devices, scientific instruments set in place before the action happens, or machines that mediate the dialectic of resistance and accommodation. [. . .] Apparatuses have no inherent ‘outside’ boundary. This indeterminacy of the ‘outside’ boundary represents the impossibility of closure [. . .]. Apparatuses are open-ended practices” (Barad, 2003: 816).

This understanding of the apparatus is one conceptual tool that allows RS technologies to be grasped in their *relation with* rather than in *opposition to* bodies. Barad’s work on the apparatus and “intra-action” (as opposed to inter-action) puts emphasis on forms of entanglement with techno-scientific phenomena that not only complicate the roles of location and distance, but also open a critical space for grasping the complicated phenomena of bodies’ extended devices of perception and sensing. It is exactly for that reason that Barad has been taken up as a reference for feminist data visualization in terms of a disavowal of the binary between body and world (D’Ignazio and Klein, 2016). Distinguished from what has been criticized as a “naturalizing” gesture in ecofeminism, feminist philosophers of science such as Barad contribute to views on proximity that surpass geographical notions of distance.

Such approaches offer feminist GIS and RS researchers a conceptual toolkit for rethinking ideas of distance. Examples of feminist critical practices using RS are indeed wide ranging. Bergmann and Lally have argued for *geographical imaginations systems* (gis), allowing for a combined visualization of both measured and experienced distance (Bergmann and Lally, 2021). In their collaborative paper, Engelmann, Dyer, Malcolm, and Powers write on “open weather,” a feminist artistic project with DIY satellite ground stations, encompassing “a series of how-to guides, critical frameworks and public workshops on the reception of meteorological satellite images using free or inexpensive amateur radio technologies.” The project takes interest in “how speculative feminist practices can transform the collection and production of images of our planet, and in doing so, produce counter-imaginaries of the climate crisis.” Taking issue with planetary images as visions of wholeness translating into ideas of fundamental distance, “so often mobilised for totalising discourses of unity in the fight against climate change,” the authors “explore how alternative earth-images can mediate local conditions, express uneven relationships to environments and facilitate moments of intimacy between strangers” (Engelmann et al., 2022: 238–239).

As toward the end of the 1990s, Litfin could write that feminist critiques of RS emerge from the “possibility [of drawing] a less sharp line between humans and nonhumans, nature and culture, men and women” (Litfin, 1997: 43). Litfin, and others after her, understand this possibility as the basis for emancipatory and integrative forms of feminist critique of RS. These reflections also give rise to reconsiderations of the possibly emancipatory potential of knowledge gained from afar as a more sustainable way of “knowing” and “caring.” The integration of the criticism of RS and GIS into its practices is particularly explicit in agendas such as the cyborg manifesto for GIS (Schuurman, 2002); feminist data visualization in GIS, using mixed methods for contextualized cartographic narratives (Knigge and Cope, 2006; Kwan, 2002); critical mapping practices of the “feminist mapping subject,” proposing feminist GIS beyond a seemingly “oxymoron” for practices of mapping that experiment with “feminist ways of looking” or ‘emotional geographies’; as well as to turn the eye to mapped relationships between, for example, gender, employment, and childcare availabilities (Pavlovskaya and Martin, 2007; Rose, 2004). As Elwood and Leszczynski show, the production of gender in the omnipresence of new spatial media differs from “GIS-era concerns,” which is why feminist approaches are demanded to respond to new practices of data creation and curation, new affordances and new digital spatial mediations of everyday life in the light of increased web-based practices. These range, for example, from various gendered uses of Open Street Map, regional dating applications, projects such as “Harrassmap,” to numerous other co-developments of gender identity and embodiment in the uses of digital geographies (Elwood and Leszczynski, 2018; Leszczynski and Elwood, 2015; Stephens, 2013).

Furthermore, feminist approaches have been incorporated into different forms of feminist data visualization, which are deeply interconnected with critical mapping practices and participatory design strategies (D’Ignazio and Klein, 2016). These also have significant influence on recent agendas of “critical remote sensing,” with the task of “exposing injustices,” “engaging situated knowledges”; and “empowering marginalized actors” (Bennett et al., 2022). Besides the vast amount of examples of the usage of RS for environmental concerns, the extension of RS skills to members of marginalized communities is seen to hold the promise of survival, empowerment, and care, like in the case of a collaboration of local institutions and communities with Swedish Forest Agency, or the use of drones by Indigenous communities in Central and South America (Bennett et al., 2022; Paneque-Gálvez et al., 2017; Sandström, 2015).

What these approaches in their various forms suggest is that RS as used technologies stand in a crucial relationship with feminist questions, but are not “ontologically” bound to what is considered as masculinist ways of distanced and detached perceiving. Like others who continue to negotiate the omnipresence of RS technologies in everyday lives (Leszczynski and Elwood, 2015), Schuurman is an important reference arguing against the critique of GIS as masculinist per se, when she claims that “[t]here is a preconception that technology is inherently masculinist. I argue that it is as masculinist as we allow it to be” (Schuurman, 2002: 261–262). The technologies’ situatedness calls for a constant questioning of the exclusions and biases they carry with them in terms of class, gender, and race, and other vectors of domination.^7^ The amalgamation of “critique” and “remote sensing” attempts to demonstrate that there is no necessary or fundamental opposition between them. In these recent approaches, feminist concerns of situatedness facilitates critiques on the level of curation of data, epistemology, as well of infrastructures of the practices of RS.

Let me briefly unpack *how* these approaches confront or integrate feminist critiques. While one theme in the literature is the mapping of issues that are of particular feminist concern—such as the lives of women, underrepresented groups, care or sex work—another is to similarly tackle epistemological problems of access and distance as method (Biemann, 2002; D’Ignazio and Klein, 2016; McLafferty, 2002; Paneque-Gálvez et al., 2017; Pavlovskaya and Martin, 2007). Epistemological arguments of feminist critiques have to be seen in co-development with more general critiques of GIS and RS. For example, Catherine D’Ignazio and Lauren F. Klein’s proposal of Feminist Data Visualization makes explicit that feminist approaches do not only concern gender, and develop an agenda to “Rethink Binaries, Embrace Pluralism, Examine Power and Aspire to Empowerment, Consider Context, Legitimize Embodiment and Affect and Make Labor Visible” (D’Ignazio and Klein, 2016). As will be shown particularly in the following section, the relation between RS and feminist critique therefore *also* takes place as a negotiation of epistemic questions of distance. Lisa Parks, for example, under the heading “plotting the personal,” explores “GPS as a means of articulating the politics of location or positionality.” In explicit consideration of feminist perspectives, she understands satellites as interactive sensors, not only as “distant relay towers in outer space” (Parks, 2001: 211). With this view on RS and initial (and partly still appreciated but more often criticized) tropes of the “ground,” we can see a rehabilitation of distant viewing taking place, yet in explicitly situated understandings.

## “I need to see it with my own eyes”: Feminist contestations of the necessity of proximity

Over the past few decades, a further tension within feminist critique has emerged with respect to an epistemically violent approach to “nature” in the light of distance. We have seen that earlier feminist critiques of RS technologies argued for the danger of “violence to nature” via their totalizing gaze. The question of violence through distanced detachment to environments remains prominent today and has in fact influenced feminist geography itself; yet one can also observe that the current focus of feminist critique has shifted and is responsive to yet another set of problems concerning field work and access (Bruun and Guasco, 2024; Guasco, 2022; Rose, 1992). The role of access, mobility, energy use, and CO_2_ emissions is increasingly put into question in the light of climate change, which affects the critique of RS both in terms of the emissions it reduces and facilitates, and the footprint produced by its infrastructures.

Celebratory discourses of RS might underplay the latter aspect and stress that the data it generates could be used to solve all such climate change-related problems. Data gathered through RS is indeed an indispensable source for climate science and various knowledges on climate change (Lintz and Simonett, 1976; Uwera, 2019). Understanding RS as part of ecologically driven research is also reflected in public discourses: for example, the Corona satellites which used to “spy on Soviet Atomic bombs” are now seen as helping to understand “ecological mysteries,” emphasizing that the “eyes in the sky” are necessary to understand the broader impact of climate change (Renault, 2021). On this view, RS significantly decreases the need to travel somewhere to gather information about a place, thereby lowering emissions. In addition, remotely sensed data is increasingly available free of charge, which democratizes access to once clandestine information. However, there are many aspects that complicate the issue of remoteness being more environmentally friendly and politically healthy, resonating with Litfin’s earlier mentioned skepticism toward overly celebratory approaches to RS (Litfin, 1997).

The footprint of satellite ground stations as well as the orbital debris produced by satellites is often underacknowledged and “invisible” factors when it comes to assessing the costs of RS (Bennett et al., 2022; Hunter and Nelson, 2021). That means, RS technologies are from an infrastructural point of view neither remote in the sense of “nowhere” nor carbon-free—their emissions are rather scattered and displaced. Furthermore, especially when it comes to RS of environments in the Global South, researchers have stressed the potential harms caused by procedures of asymmetrically “exposing” data (D’Ignazio and Klein, 2020: 105) or “othering” social groups when approaching sites in crisis, which applies to both state operators and nongovernmental organizations (NGOs) Global North situated (Rothe and Shim, 2018). These critiques of RS neither reject RS as a whole, nor do they contribute to a simple technosolutionism; rather, the engagement with infrastructures and impact of RS presents a critical angle that integrates both conceptual and practical concerns of distance.

In this context, I would like to draw attention to a line of feminist criticism that has taken the earlier critique of masculinism in a new direction, and which, in fact, has *also* made a virtue of distance. By focusing on ideas of violence and access, a certain feminist critique is concerned not with the violence of “the view from above” but rather with *the necessity to be close* to its subject of experience, which involves particular ways of allowing oneself access to and immersing oneself into an environment. This line of feminist critique—especially when it comes to that of traditional geographical research “on the ground” in general—seems to work in favor of RS technologies. Several critiques have developed Gillian Rose’s claim that the geographer’s job to go somewhere, observe, and describe has been traditionally implicated with a masculinist way of observing feminized landscapes (Rose, 1992). Following this line of thought, we can note first that, following Pavlovskaya and Martin, the “effect of feminism on geography and GIS is to not only produce gender as an object of analysis but to transform knowledge itself” (Pavlovskaya and Martin, 2007: 596–7).

What is more, putting the fieldwork paradigm^8^ of geographical research more generally into doubt results in a skepticism of the “need to always go there” (Guasco, 2022; Howkins, 2010). Research sites are often only available to the masculinist-explorative, non-disabled subject without significant care duties, excluding often those who do not meet the requirements to “go.” During the COVID-19 pandemic, for some human geographers, the necessity of fieldwork and its “contextual richness” became even more evident (Kuus, 2023). However, from another viewpoint, in-person fieldwork was revealed as being not as indispensable as is commonly presumed, contrary to disciplinary norms. Anna Guasco notes that RS data were increasingly used by geographers during this period without this, however, resulting in a sustainable integration of RS options in geographical research after the constraints imposed by the pandemic (Guasco, 2022: 469–470). Guasco problematizes a form of relation implicated into the “access” to a site: it can mean both “love” and “violence,” and the assumption to think that “assuming that every place is and should always be accessible to you” not only reveals itself as a toxic masculinist framework, but also demands, for Guasco, an “ethic of not always going there.” “Remote research and engagement are not a neutral backup, nor are they without their ethical problems. There is no neutral ground to stand on, no view from nowhere—whether you pursue research ‘on the ground’ or ‘from afar’” (Guasco, 2022: 472). In this sense, the idea of the “field” or what doing “fieldwork” means can be productively extended, innovated, and critically challenged with RS options from approaches connected to feminism, disability studies, and post-colonial theory (Bruun and Guasco, 2024).

Recent feminist critiques of the production of site-specific knowledge have reappropriated paradigms of remoteness and technology-based ways of seeing. As I have tried to point out in this section, what they question is not primarily the “view from nowhere”—the positivist and masculinist biases of science and a technologically mediated and “abstract” gaze—but the masculinist direction of research agendas in the usage of technologies, as well the masculinist underpinnings of uninhibited access. In question is also the masculinist authoritative voice of the truth coming from the “ground” or the field in terms of firsthand experience, in which the researching subject is situated in proximity to its research object, has granted itself access to it, and is embodied and situated within the “field.” Recent feminist critiques integrate earlier criticism and push back against a generalized metaphysics of presence, yet also hold a nuanced stance on how and where masculinism appears when it comes to sensing remotely.

Changes in the fundamental coordinates of feminist critiques and their particular relation to RS are not a one-way street: just as discursive critiques have been integrated with the actual practices of RS (Bennett et al., 2022; Walker, 2020), so too have changes in RS technologies pushed feminist critique to respond and develop in turn.

### Imbrications of the near and the far—keeping the actual and diminishing the felt distance?

The complex relation between distance and proximity is a defining theme in approaches to RS and its effects. Re-establishing a simplistic opposition of intimacy and distance seems counterproductive in a context where these technologies and their affective qualities in fact undermine such an opposition (Kaplan, 2017; Kurgan, 2013; McCormack, 2010). Feminist concerns with distance can indeed be used to grapple with complexity of such notions of the near and the far, which RS both reproduces and tests.

Laura Kurgan evokes this imbrication of the near and the far by describing RS as a “technology of looking close up at a distance” (Kurgan, 2013: 20–21). Relatedly, Derek P. McCormack reflects on affective and temporal dimensions and describes RS as “not so much as a technology of distanced, elevated image capture but as a set of mobile and modest techniques through which affective materials are sensed without direct contact or touch” (McCormack, 2010: 641). Caren Kaplan (2017) follows up on that thought when she claims:

The conventional binary between distance and proximity and its related oppositions—objective and subjective, global and local, unfamiliar and familiar, strange and intimate—may be culturally and historically specific to Western modernity, but even within that narrow register of human experience, there is ample evidence of greater nuance and possibility than these bluntly contrasted extremes. (p. 22)

Intertwined notions of distance and proximity do not complicate the relationship between these terms for the sake of it; rather, feminist scholars perceive this imbrication as a possible means of challenging notions of objectivity and, relatedly, objectification. Feminist approaches make a strong case for such nuanced considerations of proximity and distance with respect to RS technologies, such that, for example, “the drone challenges us to understand how that cyborg relation has become increasingly intimate, while at the same time, the proximity between the body and the machine can be ever more distant” (Feigenbaum, 2015: 282). Furthermore, what is put into question is the opposition between “only” observing on one hand, and empathically caring on the other. Traces of such ambivalences can be read in what Elizabeth DeLoughrey has characterized as “satellite planetarity.” In a combination of Denis Cosgrove’s concept of the Apollonian eye and Gayatri Chakravorty Spivak’s idea of planetarity, DeLoughrey suggests satellite planetarity as a “complex narrative legacy of modernity” that conveys “an understanding of the planet as alterity in which human vision is both connected to and disconnected from the earth” (DeLoughrey, 2014: 265–266). Furthermore, in particular stressing an alterity that does not objectify, Litfin proposes in resonance with Keller’s notion of dynamic objectivity that “Earth remote sensing could approach nature with a sense of empathy and respect, rather than as an object of planetary management” (Litfin, 1997: 42).

## Conclusion: Reappropriations of physical and critical distance in feminist critiques of RS

This article has argued that the relationship between distance and proximity in the production of knowledge, sense-making, and the manifestation of relations with the natural world is rendered increasingly intertwined in recent feminist approaches to RS and GIS. This has opened a critical space in which several concepts are on shifting territory. Integrating marginalized and feminist viewpoints into the practice of RS has been demanded and proposed (Kwan, 2002; McLafferty, 2002; Pavlovskaya and Martin, 2007), especially in the light of a notably high number of (white) male researchers doing the work and data collection of RS (Blake et al., 2021; Joyce et al., 2022). With the rise of new geographies of digital capitalism, surveillance, and knowledge production (Bennett et al., 2022), with drones (Feigenbaum, 2015) as well as “new spatial media,” such critiques prove to be an ongoing and extended concern (Leszczynski and Elwood, 2015).

What is put into question is what it means to be “critical” *toward* as well as *with* RS from a feminist point of view. I have shown that researchers are problematizing feminist critiques themselves from feminist perspectives; that they are seeing problematics of masculinism in the context of access and infrastructure; and that they are reevaluating technology-mediated modes of seeing and imagining as not ontologically bound to a gendered perspective. This shift does not only have to do with a pushback of ecofeminism by cosmopolitan “third-wave feminism” by way of identifying the former as a “touchy-feely,” religious, essentializing and backwards oriented feminism (Thompson, 2006); it crucially has to do with a re-evaluation of physical distance in its relationship to access and care, which has been significantly intensified by the COVID-19 pandemic. RS is, then, an important subject for feminist critique’s confrontation with constantly evolving practices of technology: feminist approaches to RS not only interrogate the earlier mentioned division of labor between masculine knowing-from-distance and feminine caring-in-proximity, but also offer its precise dissolution through a problematization of care(-lessness) from both near and far.

What is philosophically striking is the difference between criticizing the “view from nowhere” through the metonymy of the “ground” on one hand; and on the other hand, a critique that does not accept the nowhere-ness of the “view from nowhere” and situates, contextualizes the satellite’s perspective, “grounding” it in altogether more metaphorical sense. Furthermore, in reaction to masculinist research paradigms of “going there” and being physically on-site, researchers have challenged the trope of the “field” by reconsidering information gathered from the sky as an alternative and valuable way of gathering a *kind of truth* (Dodge and Perkins, 2009: 498), even if it is not without its own problems.

I have argued that feminist critiques can be understood as a dynamic field between theory and constantly evolving technologies and behaviors. This makes a case not only for existing impact between areas of knowledge, but also pleas for an awareness of the intertwinement of the work of physical and human geographers, RS practitioners, philosophers, and critical theorists despite considerable gaps between their practices. Rather than taking the idea of feminist critique as a one-way street where either conceptual approaches problematize technologies, or where critique can only be developed from “within” the practitioners of a technology themselves without applying “external” theoretical or abstract parameters, it seems productive to see different types of theorization in their exchange, friction, and interdependent development.

For this reason, I have advocated caution with tendencies to systematize modes of critique in terms of a normative framework of “good” versus “bad” (typically: internal/consultant/practice-oriented versus external/abstract/“only” theoretical) as to do so neglects the intellectual–historical background and epistemic coordinates of feminist critique. Such a distinction also risks neglecting a longstanding tradition of discussing “critique” within critical theory more broadly: discussions of “immanent critique,” the outside, and critical distance are found across the works of Kant, Hegel, Marx, the Frankfurt school, and a myriad of more recent contributions continuing work on the notion of “critique” or “criticality,” which cannot be excised from the term “critical.” What is more, we have seen that seemingly abstract critical reflections on concepts of nature, the human body, or objectivity have had concrete impacts on RS and GIS. The heated debates between GIS practitioners and critics in the 1990s can be said to have given rise to participatory GIS, voluntary GIS, and more broadly feminist concerns within RS practice.

Particularly in response to both global climate change and to masculinist underpinnings of the traveler–explorer research subject, RS is now seen in a more ambivalent light than was often the case in the 1990s. In this sense, feminist critique has notably reappropriated the concept of distance. Still, it might be asked, with the grain of those earlier critiques: have recent feminist discourses effectively bought into an “unconscious assimilation of cultural moods” (Garb, 1990: 276) or into the “masking function” (Litfin, 1997: 29) of celebratory discourses of RS? Not quite. Rather than claiming that recent feminist approaches tend to celebrate RS technologies and leave behind what had been articulated as their critique, it seems that the direction of problematization has shifted.

Feminist critique has not lost its critical potential, but rather has redefined its coordinates. This has to do with developments of RS technologies themselves, with alternative theoretical frameworks for approaching sensing bodies, technology-mediated relationships with the natural world, and the continuously developing theme of objectivity in respect to climate change research. What remains in more recent forms of critique is as insistence that, for example, that remotely sensed data’s objectivity has to be put into the context of its own (un)certainty and the infrastructures of production and access. Access to both field sites—the vaunted “ground” of RS—and to data therefore turns out to be one of the core arguments of feminist approaches. Rather than identifying technologically remote views with ontologically inscribed gender inequalities, recent feminist critique seems to put stronger emphasis on infrastructural critique.^9^

I have furthermore drawn attention to the role of imagination in more recent critiques of RS and GIS, which often stem from researchers with feminist approaches. Examples include the suggestion of geographic imaginations systems (gis) (Bergmann and Lally, 2021), the public participation project “open weather” (Engelmann et al., 2022), as well as historical inquiries into RS that shed light on imaginaries and “malleable and uneven perception of worlds” (Kaplan, 2017: 216). Presence and loss are made increasingly visible through RS, and while continuing to scrutinize and problematize ways of looking, feminist critique has also reclaimed the concept of distance, particularly with respect to caring-across-distance.

One approach to the separation of critics and practitioners of RS would entail a plea to “close the gap” between them. However, I would rather see this gap as demanding a search for difficult and heterogeneous transitions between the forms of critique I have discussed, and therefore suggest a strategy of “coping with the gap.” This concerns particularly the different levels of feminist critique, some of which are straightforwardly theoretical, whereas others are directly practice-oriented. Considering the debates and criticisms of feminist critique of RS and GIS in the 1990s, I would caution against the demand that all critique speak directly to the practice or give concrete advice to it. Rather, it seems preferable that it speaks in different registers, taking up concerns as various as developing data literacy, increasing points of contact, or providing a critical mediation to shine a light on the relationship between data, images, and questions of care.

## Notes

1. Definitions of remote sensing (RS) take as their starting point the “acquisition of data of an object without touch or contact” (Lintz and Simonett, 1976) or “worldly apprehension without the immediacy of touch” (McCormack, 2010: 642).

2. RS and Geographical Information Systems (GIS), as Bennett et al. point out, “remain two distinct (if sometimes overlapping) disciplinary fields that have motivated different inquiries,” they also “share intertwined origins” (Bennett et al., 2022: 731). Acknowledging the differences of RS and GIS, I look at them jointly, for the reason that feminist critiques share similar concerns when it comes to distance, objectivity, and masculinism.

3. Here, I would like to point to Thompson’s analysis that ecofeminism has been relegated as a marginal position in academia, in ways that sometimes would attempt to overcome ecofeminism at the cost of tendentially dismissive views of local forms of living and reproductive labor, and to therefore “throw out the baby with the bathwater.” Against this, Thompson argues, a rereading of some of the main ecofeminist attempts in combination with third-wave feminism would strengthen the field of feminist critique (Thompson, 2006).

4. This problematized parallelism of the feminine, motherhood, and nature, is perhaps most poignantly described by analogisms between being on Earth and being inside a womb (Garb, 1990: 272–273).

5. As Tollmann shows, despite some interesting crossings in Arendt’s criticism of space flight, Arendt and Haraway’s arguments mobilize different ideas of the human (Tollmann, 2022: 249–251).

6. The contextualization of RS and combination with the knowledge of local stakeholders, for example, has been pioneering work that arguably resonates with feminist critiques, even though it might not necessarily originate from a feminist framework (Robbins, 2003; Robbins and Maddock, 2000).

7. The lacking interconnection of gender, class, and race has also been lamented as a silenced concern in the research of feminist digital geographies, which over the past decade, however, witnesses more sustainable engagements with intersectional agendas (Elwood and Leszczynski, 2018).

8. It is a certain skepticism toward RS as a “quantitative method” that has led to its unjustified dismissal. In this sense, Jiang argues, RS analysis “offers additional stories about changes in the landscape—stories not told by interviewees, or stories that supplement the account of interviewees.” (Jiang, 2003: 215).

9. For this notion of infrastructure and infrastructural critique, the term’s historical development, conceptual underpinnings, as well as a recent increase in usage has to be considered (Kroth, 2022).
